# Trypan Blue Image-Guided Removal of Surface-Based Bacterial Biofilms from Chicken Tissue Using Cold Atmospheric Pressure Plasma

**DOI:** 10.3390/plasma8030034

**Published:** 2025-08-26

**Authors:** Michael Okebiorun, Dalton Miller, Kenneth A. Cornell, Jim Browning

**Affiliations:** 1 Biomedical Engineering Program, Boise State University, Boise, ID 83725, USA; 2 Department of Chemistry and Biochemistry, Boise State University, Boise, ID 83725, USA; 3 Department of Electrical and Computer Engineering, Boise State University, Boise, ID 82725, USA

**Keywords:** atmospheric pressure plasma, image-guided treatment, bacterial biofilm, wound, debridement

## Abstract

The study evaluates the efficacy of an image-guided CAP treatment method with a plasma device capable of rapid biofilm removal from chicken tissue. The plasma treatment operating configuration includes a gas mixture of Argon and H_2_O at a flowrate of 1.5 lpm. An X-Y stage was used to move the chicken sample below the stationary plasma scalpel at a speed of 0.1 mm/s. The discharge voltage and current were maintained between 3.2 and 3.7 kV (AC 20 kHz), and at 3 mA, respectively. The electrode gap and sample distance were set to 0.6 mm and 4 mm. This configuration facilitated effective biofilm removal, as confirmed by CFU analysis and 3D microscopic analysis showing a >99% reduction in biofilm post treatment with an etch rate of 2.2–5.8 μm/s and an impact width of up to 300 μm. The plasma scalpel electrode temperature reached 94.7 °C, while the targeted biofilm area was heated to 36.3 °C, suggesting non-thermal biofilm disruption. Three-dimensional microscopic analysis revealed biofilm thickness on chicken tissues ranging from 20 to 180 μm, comparable to biofilm loads on mammalian tissues. In conclusion, the study highlights the potential of CAP devices as a promising solution for biofilm debridement.

## 1. Introduction

Chronic wounds present multifaceted challenges in medical treatments, particularly when bacterial biofilms complicate the healing process [1–4]. A pressing issue in the management of such wounds is the difficulty in effectively imaging bacterial biofilms [5–8]. Existing imaging techniques, while promising, often fall short in clearly distinguishing biofilms from tissue-like substrates at the mesoscale level [6], which is pivotal for precise, surface-based treatments of biofilms on wound surfaces. In the quest to address this limitation, the potential of trypan blue (TB) as a selective dye for biofilm imaging stands out [9]. Historically, TB has been utilized for a range of applications, including as a staining dye in biopsies, living cells, organisms, and even as a textile colorant [10–14]. Chang et al. [15] leveraged TB in an image-guided surgery study, revealing that a 0.1% concentration of TB was adept at staining a rabbit’s anterior capsule, though other studies have varied in their optimum concentrations. A critical mechanism of TB is its ability to permeate the compromised cell membranes of dead cells, subsequently staining cell components like DNA, RNA, and polysaccharides. Given the distinctions in factors such as lipid membrane composition and transmembrane potential between mammalian cells and bacterial biofilms, there is a growing anticipation that TB could be uniquely suited to selectively stain bacterial biofilms on wound surfaces with an optimized concentration and staining period. Our recent study showed that TB staining at 0.04% concentration and 10 min staining period was able to obtain segmented images of bacteria biofilms on chicken tissue, with images taken under purple and cyan lights [9].

However, the visualization of biofilms is only one part of creating an effective surface-based treatment of bacterial biofilms on chronic wounds. A biofilm removal method, debridement, is also required. The intricate architecture of biofilms, where bacteria are engulfed within protective layers of extracellular polymeric substances, renders them notably tolerant to both biochemical and mechanical removal methods. Cold atmospheric pressure plasma (CAP) emerges as a potential solution [16–19]. CAP, alternatively known as low-temperature plasma or simply cold plasma, is a mixture of reactive species, charged and neutral particles, and photons, all generated at atmospheric pressure and near-room temperatures [20]. CAP’s recent rise in biomedical applications is underpinned by its proven anti-microbial capabilities, along with reported efficacy in other medical arenas like cancer treatments and even hair treatments. In cancer treatments, CAP selectively induces apoptosis in cancer cells by generating oxidative stress while sparing healthy tissue. This has been particularly effective in melanoma treatment, where both direct and indirect CAP methods have shown significant reductions in cancer cell viability. For hair treatments, CAP has demonstrated the ability to stimulate the proliferation of dermal papilla cells, which are critical to the hair growth cycle, promoting hair regeneration [21]. CAP has also been used in physical treatments such as a plasma comb to neutralize pathogens and potentially aid in hair follicle recovery [21–25]. What distinguishes CAP further, aside from its potential therapeutic abilities, is its documented safety profile positioning it as a potentially transformative tool in the fight against chronic wounds complicated by bacterial biofilms [26–28]. In our prior work [29], we demonstrated biofilm removal from chicken tissue using autofluorescence guided X-Y stage to selectively remove the biofilm with our plasma scalpel device. While that work demonstrated biofilm removal, it was limited to use with auto-fluorescing biofilms. Here we combine our TB imaging method with our plasma scalpel device. This current study heavily relies on the methodologies and findings from our previous works [9,29], where readers can find additional detailed characterizations of biofilm imaging and removal processes, such as optimized staining protocols, multi-colored LED lighting parameters for differentiation (e.g., cyan ~ 500 nm and purple ~ 400 nm with image subtraction techniques), fluorescence validation standards, and plasma device operations under autofluorescence guidance, to further understand the integrated approach presented here.

In this study, CAP, for the first time to the best of our knowledge, was used to effectively remove bacterial biofilms from chicken tissue (wound model) guided with trypan-blue based imaging. Biofilm maps obtained from optical imaging of TB-stained chicken biofilms were utilized to guide the CAP treatment with a motorized X-Y stage and a plasma discharge apparatus (plasma scalpel) to create a plasma debridement system [30]. The CAP setup was characterized with specific treatment parameters, including power settings (discharge voltage of 3.2–3.7 kV at 20 kHz and current of 3 mA), gas flow rate (Argon mixed with H_2_O at 1.5 lpm), treatment distance (4 mm from sample to electrode outlet with a 0.6 mm electrode gap), and stage movement speed (0.1 mm/s), to enhance reproducibility and facilitate reader understanding, building on the detailed plasma characterizations in our prior work [29]. The biofilm removal efficacy was evaluated via 3D microscopic imaging to determine biofilm etch rate and CFU count reduction analysis, in addition to previous techniques used in the previous studies [9,29].

## 2. Experimental Procedures

**Figure 1.**
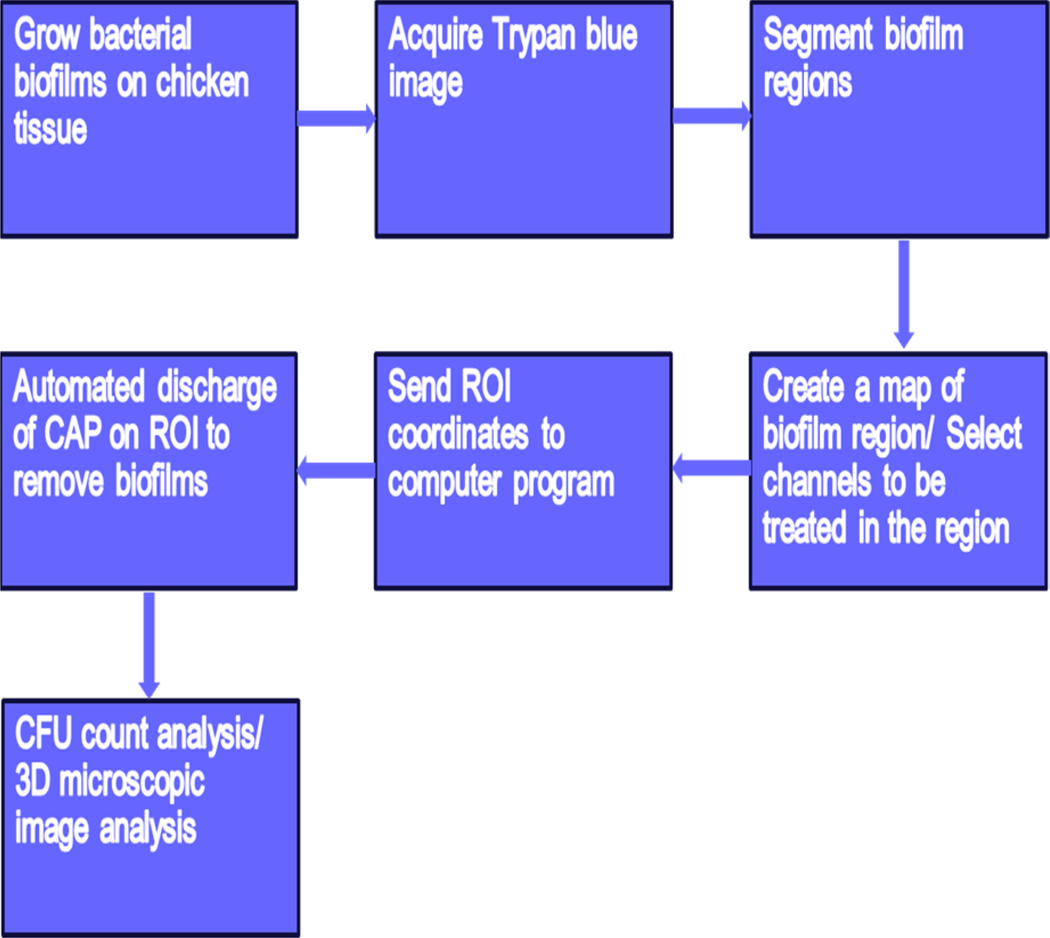
Overview of the core steps involved in the research study which includes experimental procedure as well as validation methods. The sample substrate is chicken tissue, while the fluorescent substance is pyoverdine contained in *Pseudomonas fluorescens* biofilms.

Figure 1 provides an overview of the entire experimental procedure in the imaging and removal of bacterial biofilms from chicken tissue and the validation techniques. First, bacterial biofilm is grown on chicken tissue with the skin removed. The sample is stained with TB and imaged using TB imaging method described here in our previous work [31]. A map of the biofilm is created for selective treatment, or a desired treatment channel is selected and used to guide the CAP treatment at predefined locations. Finally, either CFU counts are measured from the fully treated samples or 3D microscopic image analysis is performed using the channel treatments. These steps are described in detail below.

### 2.1. Biofilm Sample Preparation

In our study, biofilms were grown on chicken tissue, specifically sourced from fresh skinless chicken breasts purchased from a local grocery store. These breasts were carefully cut into segments approximately 1.2 cm × 1.2 cm × 0.5 cm in size and placed into a sterile 150 mm Petri dish. Each tissue piece was meticulously cleaned with sterile deionized water and then wiped with sterile paper towels moistened with isopropanol. After a brief period of air drying in a biosafety cabinet, the tissue samples were arranged in the wells of a 6-well plate. Each well contained 0.5 mL of 1% agarose in Dulbecco’s Minimal Eagles Medium (DMEM) to immobilize the tissue. A total of 12 chicken tissue samples were prepared. For inoculation, a cotton swab containing a 1:10 diluted sample of the *P. fluorescens* overnight culture in sterile LB, was applied to the center of each chicken tissue sample. The samples were then incubated for 24 h at 25 °C. The *P. fluorescens* commonly forms biofilm in the tissue as the nutrients come from the chicken allowing the bacteria to grow and form the extra-cellular matrix. For visualizing the biofilms, TB staining was performed using a technique previously determined to be effective [9]. Specifically, a 0.04% TB solution in PBS was used, immersing chicken samples with 1-day-old biofilms for 10 min [9]. Following the staining process, the samples were rinsed in PBS to remove any unbound dye. This method enabled clear visualization of the light blue-stained biofilms on the more deeply stained chicken tissue showing a difference their TB uptakes at this concentration and period as shown in our previous work [9].

### 2.2. Biofilm Visualization and Imaging System

**Figure 2.**
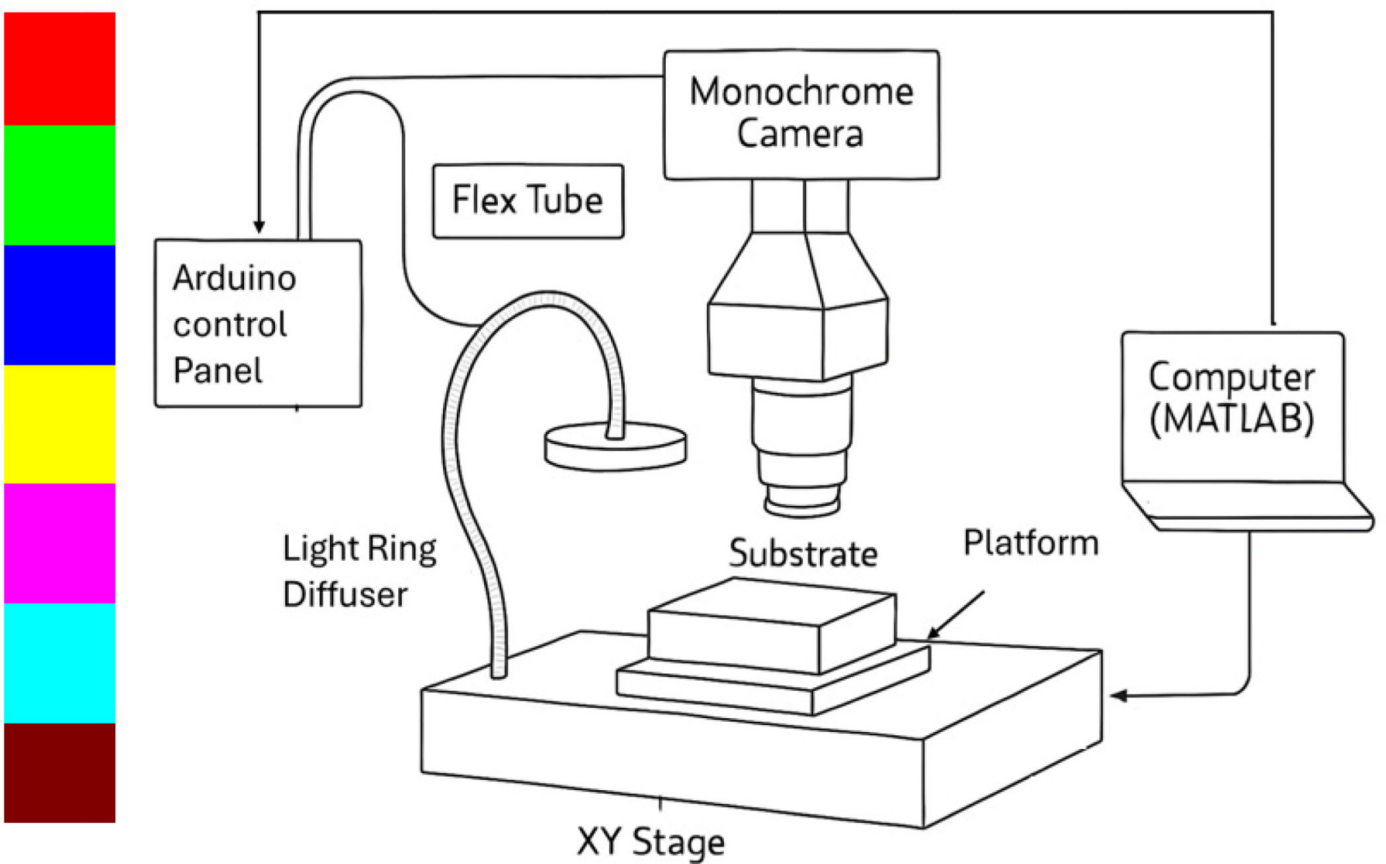
Imaging setup after the sample undergoes Trypan blue staining. Image acquisition is carried out using a Mightex monochrome camera viewing samples illuminated under various colored lights for automated segmentation of the biofilm region. (The LED colors explored for image optimization are shown on the left side of the panel).

The imaging and analysis of biofilms underwent a multi-faceted approach, utilizing TB staining and optical imaging to guide and evaluate the CAP treatment process. The primary imaging setup, as illustrated in Figure 2, comprises a Mightex monochrome camera, a Neopixel 256-LED array, a 405 nm fluorescent lamp, and an integrated control system using an Arduino Uno with MATLAB (Toolbox Release 2012b, The MathWorks, Inc., Natick, MA, USA), and LabVIEW 2021 (National Instruments Corporation, Austin, TX, USA). This arrangement facilitated the automation of image acquisition and processing. For the TB-stained biofilm imaging, specific-colored lights—cyan and purple—from the LED array were used. This choice was guided by a technique established in previous work [9] which involved the subtraction of the cyan image from the purple image to effectively segment the TB image. This segmentation technique was crucial in guiding the CAP treatment process.

### 2.3. CAP Operation and Characterization

**Figure 3.**
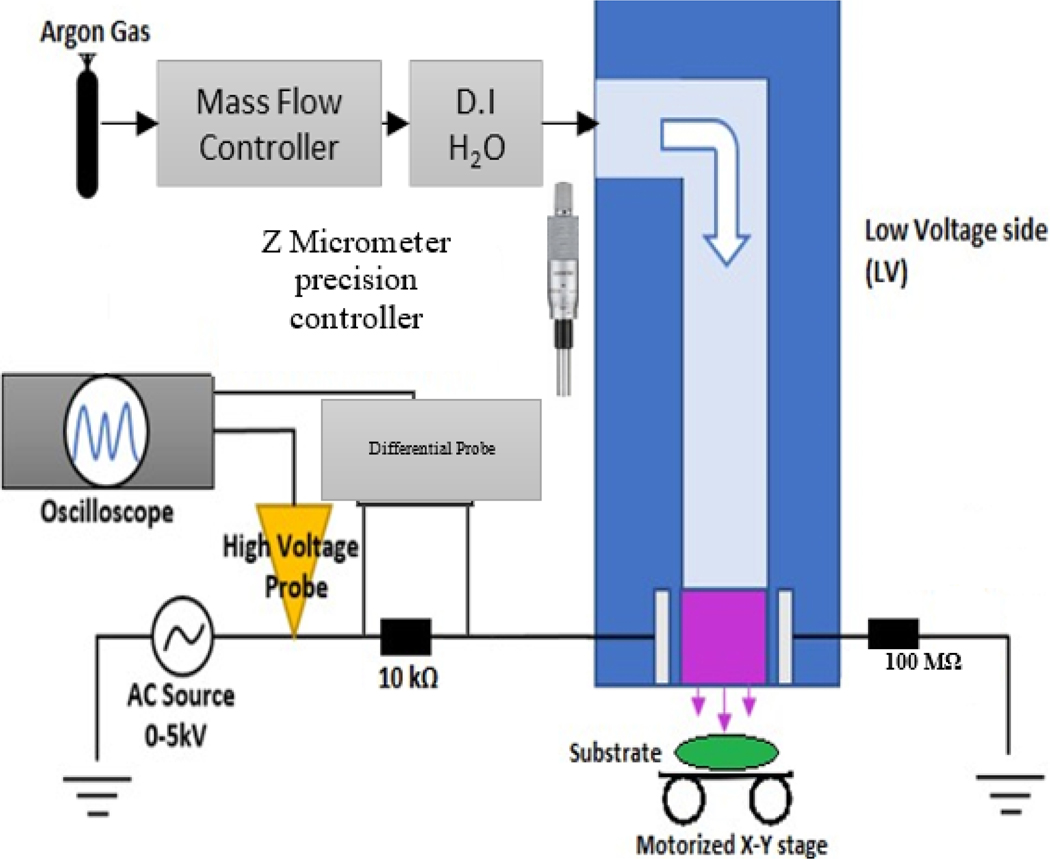
Plasma Device Schematic. Voltage and current measurements are obtained via the high voltage probe and the differential probe, respectively, with the ballast resistor incorporated in between its leads. The 100 MΩ is connected to the ground to limit and stabilize the plasma discharge. The gas is passed through water before going into the dielectric barrier discharge of the plasma scalpel device.

The plasma setup was developed and is characterized by its unique design and operational capabilities, as depicted in Figure 3. The device generates plasma by directing argon gas through a mass flow controller and then through de-ionized water in a Dreschel bottle. The gas flows between two metal electrodes covered with dielectric material to form a dielectric barrier discharge configuration. This setup, leveraging the production of reactive oxygen species from the argon-water combination, is pivotal for microbial inactivation and removal. The plasma apparatus, fabricated from low-temperature co-fired ceramics (LTCC), features a discharge outlet of approximately 1.0 mm × 0.6 mm. We refer to this device as the plasma scalpel. The device is discussed in detail here [30]. The fabrication of similar devices by our group with detailed characterization results are further discussed here [18,32]. The electrode configuration includes a high voltage electrode connected to an AC power supply (0–5 kV, 20 kHz) and a low voltage electrode grounded through a 100 MΩ resistor. The current is measured via a differential probe on a 10 kΩ resistor on the HV side, and voltage is measured using a high voltage probe. Both current and voltage measurements are displayed on an oscilloscope. These diagnostics are used to characterize the plasma including IV measurements.

Temperature measurements were conducted at both the sample and electrodes using a FLIR system ThermoVision A320 Camera (Teledyne, FLIR, Wilsonville, OR, USA), essential for assessing the thermal effects on the samples during treatment. The specimen was positioned on a motorized X-Y stage, enabling bidirectional movement and precise positioning at a speed of 0.1 mm/s, controlled via LabVIEW software (2023 version). This setup ensured careful control over the treatment area, with an electrode gap set to 0.6 mm and a sample distance of 4 mm adjustable through a Z-Micrometer for fine-tuning vertical proximity and optimizing plasma characteristics for effective biofilm eradication. The plasma was generated using a gas mix of Argon and H_2_O at a flowrate of 1.5 lpm, further fine-tuning its properties for targeted biofilm treatment. A mass flow controller, also via the central LabVIEW program, regulates the gas flow. Furthermore, for targeted treatment, we converted the identified TB bacterial biofilms’ pixel coordinates into real-world coordinates. This mapping, combined with the X-Y stage’s movement and a computer vision-like algorithm, ensures that plasma is applied precisely on the biofilm regions. Notably, while the plasma device remains stationary, emitting plasma downwards, the substrate is dynamically adjusted on the X-Y stage for optimal coverage.

### 2.4. Microscopic Imaging and CFU Analysis

To further understand the biofilm removal capacity of the CAP on biofilms relative to the background tissue, a systematic approach was implemented to etch channels using CAP, followed by a detailed analysis using 3D microscopic imaging and colony-forming unit (CFU) count reduction assessments. Four distinct approaches were employed to gauge the CAP’s spatial precision, biofilm etch rate, and effectiveness by using 3D microscopy. These included:

1. A single pass over a channel at a speed of 0.1 mm/s.
2. Two consecutive passes over the same channel, maintaining the speed at 0.1 mm/s.
3. Three passes over the same channel, again at a speed of 0.1 mm/s.
4. Creating five adjacent channels, each with a single pass at 0.1 mm/s.

Post etching, these channels were imaged under a Keyence VHX 7000 series digital microscope. This advanced imaging allowed for the capture of detailed etch profiles and the measurement of the depth of the etched regions with high precision. The objective was to compare and contrast the impact of different passes and patterns on the biofilm structure and to determine the biofilm etch rate.

In addition to the microscope evaluation, trypan blue images of the biofilm regions were obtained using the imaging setup in Figure 2 to guide CAP treatment. The TB imaged biofilms were either treated with the CAP discharge or were used as gas controls for CFU counts. This method provided a quantitative measure of the biofilm reduction post-CAP treatment. By calculating the percentage log reduction in CFUs with respect to untreated samples, we could determine the extent to which CAP treatment has removed the biofilms. To perform CFU analysis, a sterilized punch (6 mm diameter) was used to excise the treated regions from the chicken tissue samples. For the 1-pass treatment, the entire chicken surface was treated with a single pass per spot; for the 2-pass treatment, the same regions were treated twice; and for the 3-pass treatment, the regions were treated three times. The excised tissue punches were transferred into test tubes containing 5 mL sterile phosphate-buffered saline (PBS) and 10–15 glass beads (3 mm diameter) to facilitate mechanical disruption. The samples were then vortexed vigorously for 2 min at maximum speed to disaggregate the biofilms and liberate viable cells from the tissue surface, similar to methods described for biofilm removal from substrates [33]. Following vortexing, serial dilutions of the resulting suspensions were prepared in sterile PBS, and 100 μL aliquots were spread-plated in triplicate onto Luria–Bertani (LB) agar plates. The plates were incubated overnight at 25 °C, and total colony-forming units (CFU) were enumerated by counting visible colonies and multiplying by the relevant dilution factor. All treatments were performed in triplicate, and the experiments were repeated three times to ensure reproducibility. Gas-only controls (without plasma discharge) and untreated samples were processed identically to account for any non-plasma effects.

## 3. Experimental Results

### 3.1. CAP Device Characterization

**Figure 4.**
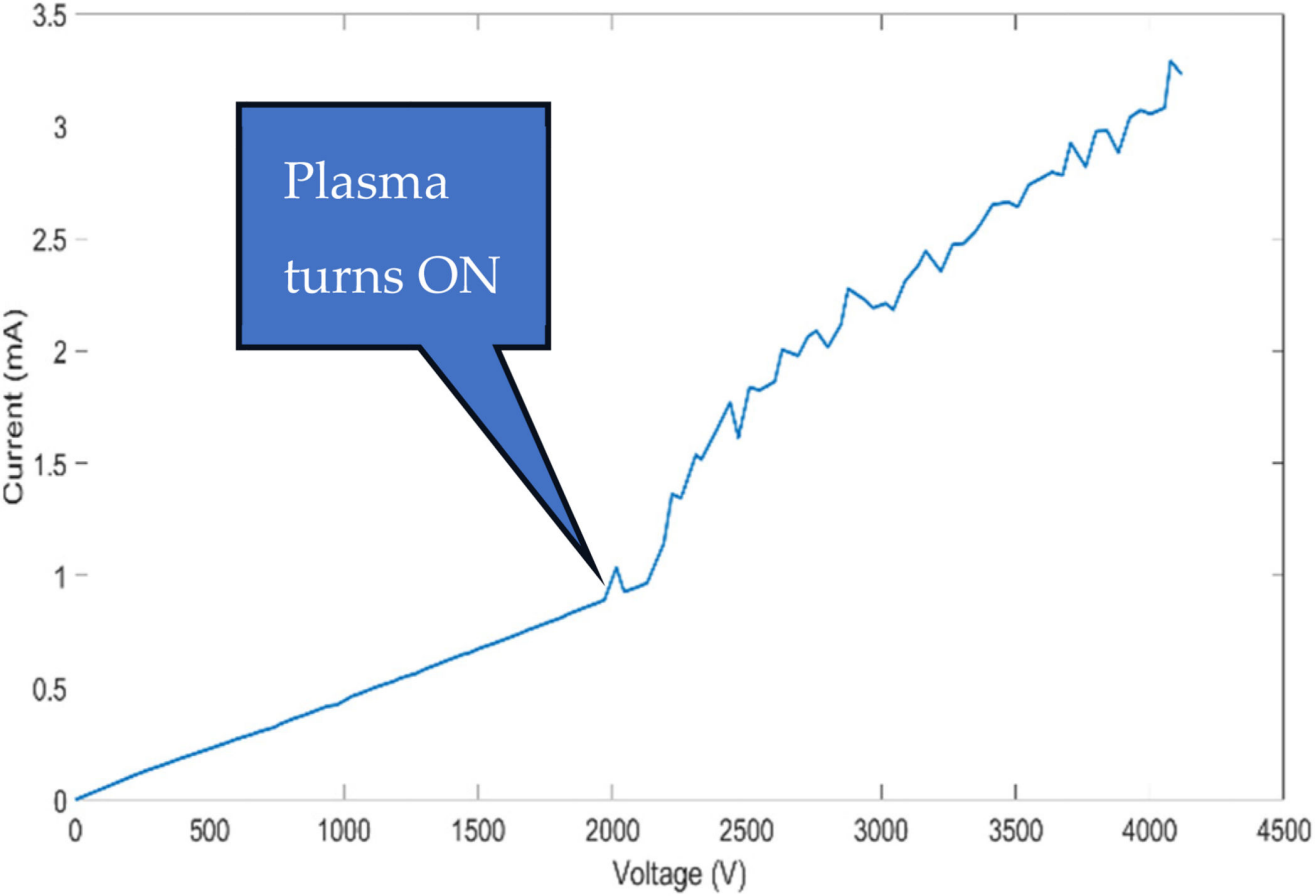
IV curve showing the electrical response during power-up. The plasma as seen here is ignited at about 2 kV, 0.8 mA; however, the plasma operating conditions can be increased to a set maximum of 4.2 kV, 3.3 mA.

The current–voltage (IV) curve depicted in Figure 4 provides an insight into the electrical behavior of the CAP device during the power-up sequence. An inflection point occurs at approximately 2 kV, where the current sharply increases, indicating the initiation of plasma. The displacement current at this point is approximately 0.8 mA. The current reaches about 3 mA at a voltage of 4.2 kV. Typically, the device was not operated beyond this point to prevent plasma scalpel damage. The roughly linear increase in current with increasing voltage suggests a relatively stable plasma density increase (discharge current) with voltage. The discharge current is the primary diagnostic tool comparing scalpel operations.

**Figure 5.**
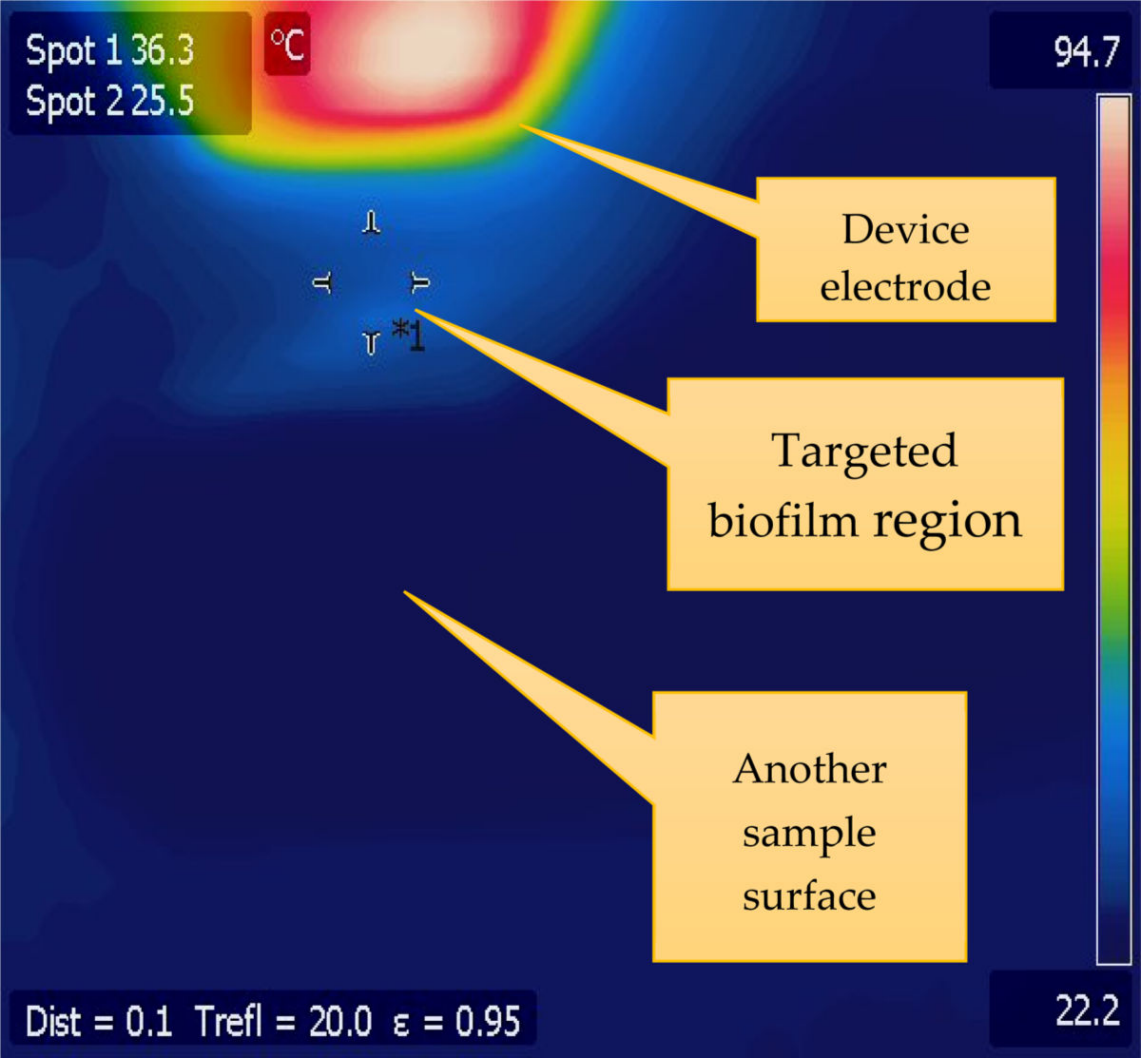
Temperature distribution obtained using a FLIR systems ThermoVision A320 Camera during plasma action. The electrodes become as hot as 94.7 °C, the targeted biofilm region is approx. at 36.3 °C, while the rest of the chicken sample remains at room temperature. This treatment was performed typically at 3.2–3.7 kV, 3 mA, 4 mm sample distance, gas mixture of Ar and H2O at 1.5 lpm and X-Y stage speed of 0.1 mm/s. The “*1” at the targeted biofilm region is just a focus marker ID for the particular spot.

Figure 5 depicts the temperature distribution during CAP device operation at 3.5 kV, 3 mA measured by an IR camera. The electrode reaches 94.7 °C—indicative of direct plasma interaction and current flow—while the targeted biofilm area is heated to 36.3 °C, suggesting potential disruption of biofilm structure was likely not due to thermal damage. The rest of the sample remains at room temperature, highlighting the device’s capability for localized treatment. It also shows that the device should be safe for use in the treatment of mammalian tissue.

### 3.2. Biofilm Reduction Analysis

**Figure 6.**
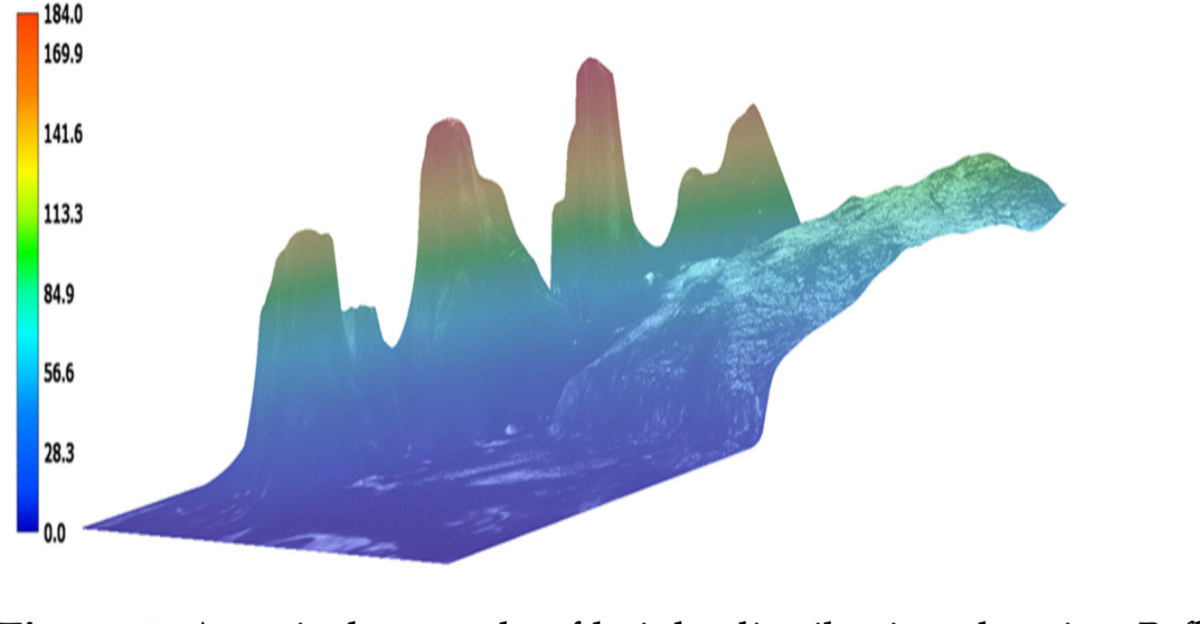
A typical example of height distribution showing *P. fluorescens* biofilms on chicken; the biofilm thickness ranges from 49 to 185 μm. This 3D microscopic imaging was guided by the TB stain on the sample. The background (0.0 μm) is the chicken background while the biofilms are the irregular shapes illustrated by the jet color variation. The y-axis (also represented as the color bar) is the height of the biofilms from the surface of the chicken.

**Figure 7.**
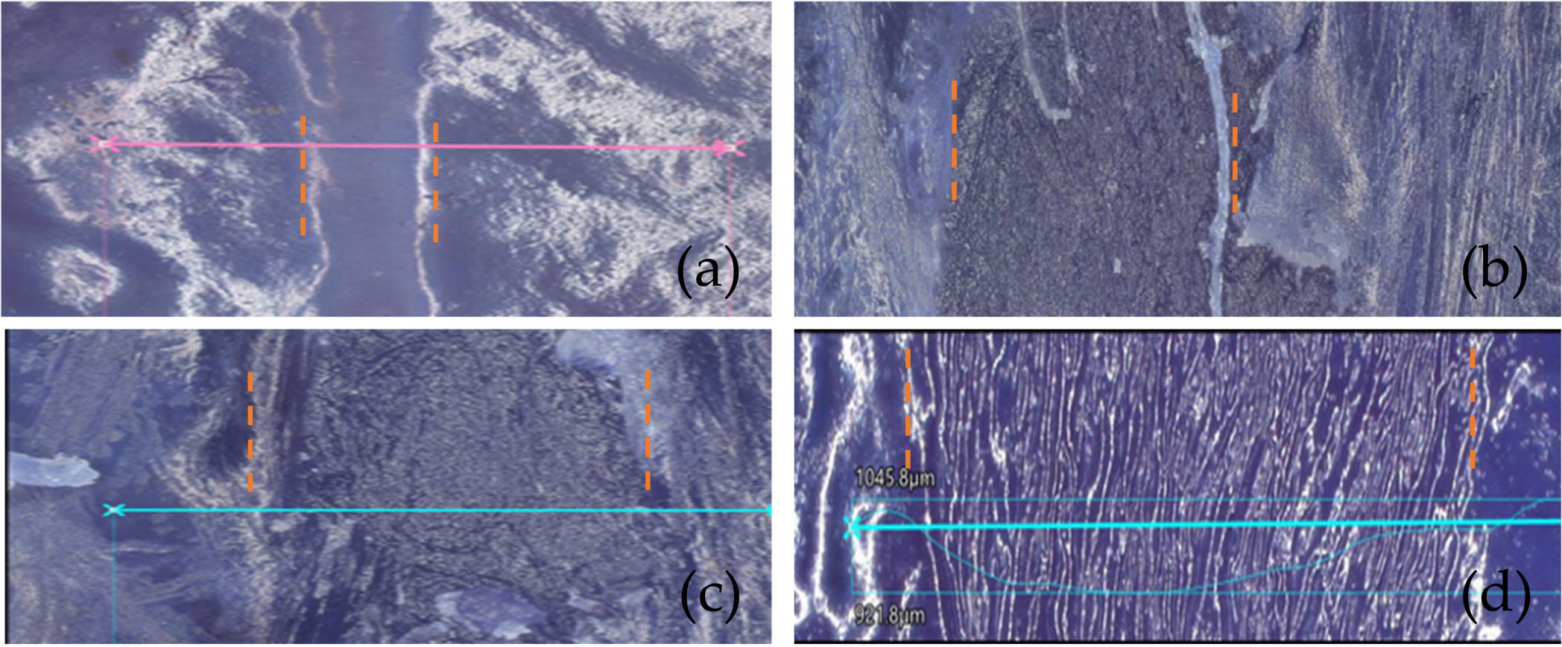
Microscopic images of etched channels for 4 different samples with the following treatment settings: (**a**) 1-pass treatment, (**b**) 2-pass treatment, (**c**) 3-pass treatment, and (**d**) 5-pass side-by-side treatment with a pitch of 100 μm between the passes. The orange traces highlight the edges of the etched channels, and the horizontal lines (blue and purple—the colors are different just based on the machine’s default setting for the current magnification) are etch profile markers from the Keyence VHX-7000 imaging software.

To provide reference for the biofilm uniformity, Figure 6 provides the three-dimensional landscape of *P. fluorescens* biofilms on chicken tissue visualized under the Keyence Digital VHX-7000 microscope (Keyence, Osaka, Japan). The biofilm regions have varying thickness of the biofilm, with heights ranging from 49 to 185 μm. The topographical variance, as highlighted by the jet color map, underscores the irregularity in biofilm formation. This may impact treatment passes such that some densely populated regions may require multiples passes to achieve complete biofilm eradication. Next, channels were etched in the biofilm samples. Figure 7 shows the microscope images of channels etched by the CAP device for 4 treatment configurations: (a) 1-pass treatment, (b) 2-pass treatment, (c) 3-pass treatment, and (d) 5-pass side-by-side treatment with a pitch of 100 μm between two adjacent passes. The variation in magnification of the images is due to attempts to obtain the optimal image for each sample. These channels are the result of targeted CAP treatment, aimed at removing identified biofilms from the tissue’s surface. This treatment was performed typically at 3.2–3.7 kV, 3 mA, 4 mm sample distance, gas mixture of Ar and H_2_O at 1.5 lpm and X-Y stage speed of 0.1 mm/s. Here the channels in the biofilm can be clearly observed. The orange traces highlight the edges of the channels. The horizontal lines (blue and purple—the colors are different just based on the machine’s default setting for the current magnification) are the profile path measurement tracks used by the microscope. Due to the TB stains on both the chicken tissue, biofilm region and treated channels, as well as the magnification level, the bacteria cells are not visible or identifiable. So, we visually inspected the biofilm region/treated channels to guide the microscopic imaging of the region. It is also worth noting that clear visualization of biofilm regions relative to non-biofilm regions have been shown in a previous study [9]. Three-dimensional analysis is, however, required to understand the extent of the effects of CAP on the biofilm/chicken tissue.

**Figure 8.**
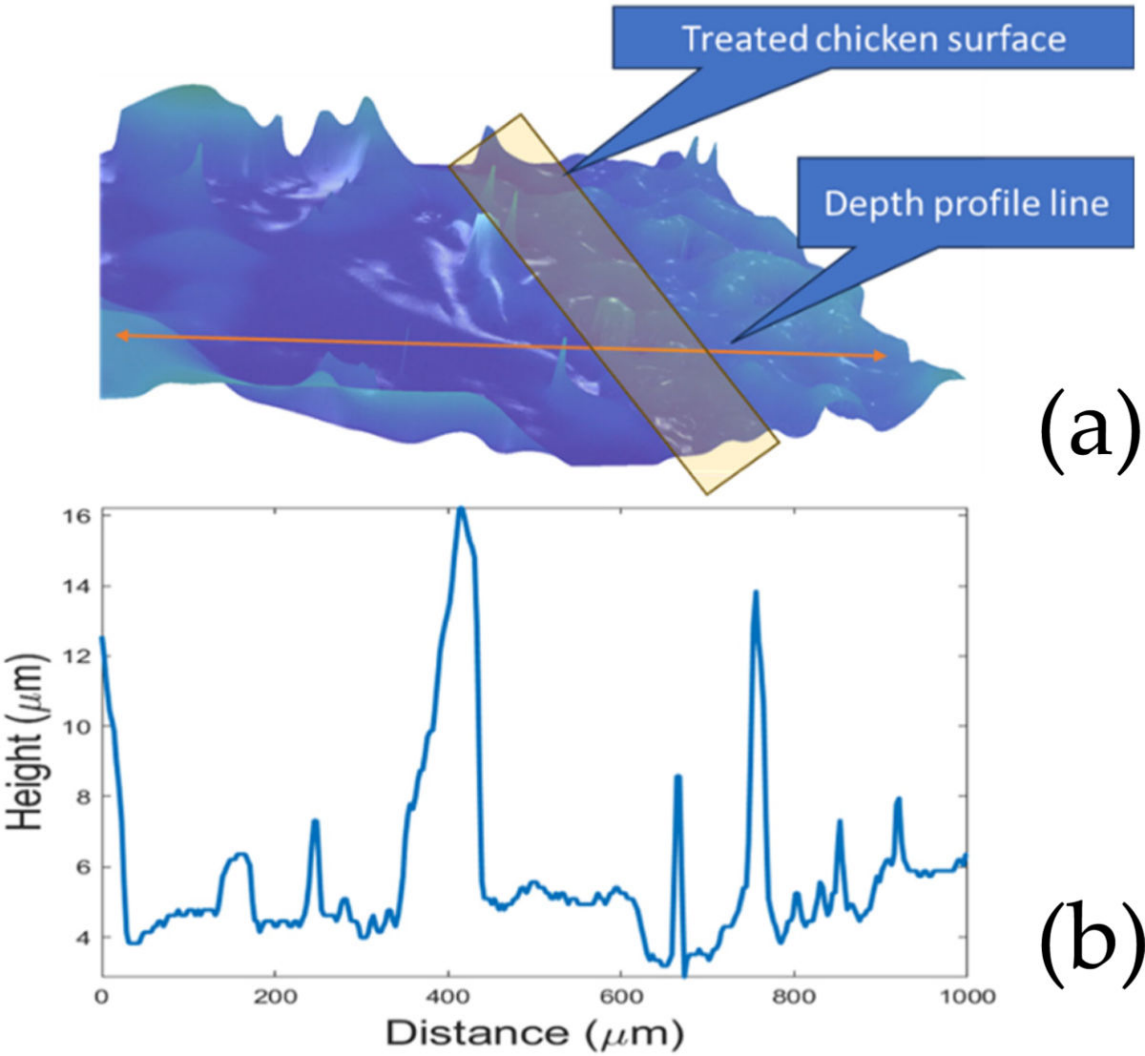
(**a**) Image of 3-pass treated chicken sample with no biofilm and (**b**) the depth profile showing the action of CAP on just chicken tissue. The height profile due to the uneven nature of the chicken surface shows a depth variation of 16 μm in the horizontal slice. No significant depth reduction is, however, observed from plasma treatment.

Next, the chicken was treated only with the scalpel. Figure 8 provides a visual and quantitative analysis of a three-pass treated chicken sample, without the presence of biofilm, to ensure that the plasma treatment does not adversely affect the underlying tissue and to help in determining the biofilm etch rate. The 3D image reveals the topography of the treated area, and the corresponding depth profile line charts quantify the surface variations. The height profile across the selected horizontal slice of the treated surface shows variations with a maximum amplitude of 16 μm. These variations fall within the natural texture of chicken tissue, suggesting no significant depth reduction or tissue damage attributable to the CAP treatment. It should be noted that the Keyence digital camera corrects for issues with tilting or bent surfaces. The irregularities are very small such that we are not able to align the depth profile line and the profile itself by visual inspection. The absence of any substantial etching or gouging post-CAP application is a positive indication that the device operates within safe parameters on tissue. It is worth noting though that the topology of the chicken surface might be construed as biofilm heights on a microscopic 3D scale; therefore, it is important to be guided with TB differentiative stain imaging. The data support the device’s selectivity in targeting only biofilm, preserving the integrity of the healthy tissue beneath. Note that this selectivity to chicken tissue also means that the chicken acts as a plasma “etch stop”, greatly reducing the etch rate. Preliminary tests, however, suggested that prolonged tissue treatment or deviation from the treatment parameters described in the method section such as not moisturizing the gas, or too short or too long sample distances can cause burns in the chicken tissue.

**Figure 9.**
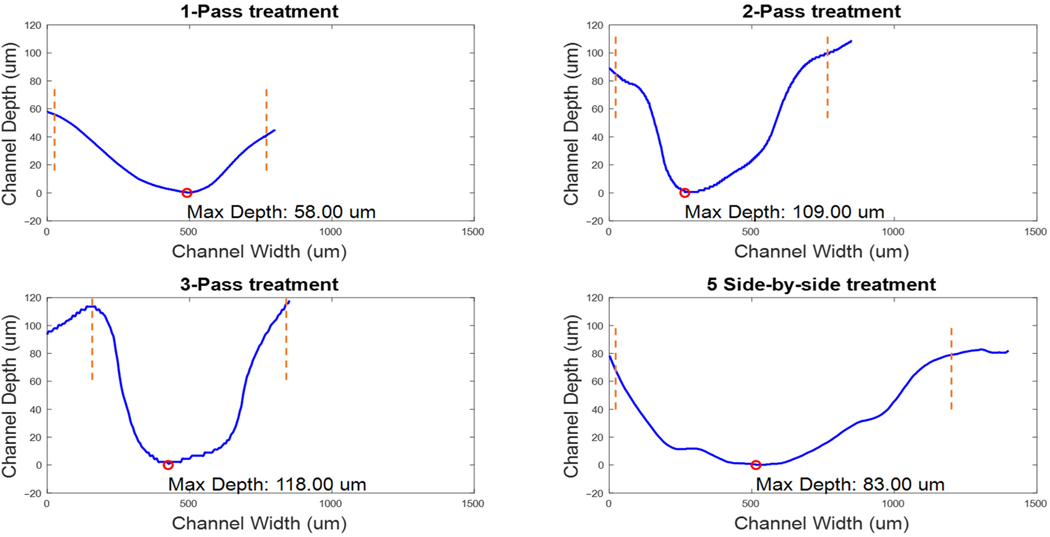
Depth profile to evaluate the amount of bacteria biofilms removed from the chicken surface. The orange traces are the bounds of the CAP treatment. The 1-pass treatment has a maximum biofilm depth reduction of 58 μm; 2-pass: 109 μm; 3-pass: 0–118 μm; and the 5 side-by-side: 83 μm.

Figure 9 shows the biofilm etch profiles of the four different CAP treatments on chicken tissue for the channels imaged in Figure 7. The different passes allow determination of the biofilm etch rate indicative of biofilm removal efficiency. The etch rate is calculated by determining the effective plasma exposure time. For a 1 mm discharge length and a movement speed of 0.1 mm/s, any biofilm location is exposed for ~10 s. Dividing the measured etch depth by this exposure time provides the etch rate. The single pass treatment shows an etch rate of 2.2–5.8 μm/s (minimum etched depth and maximum etched depth), offering a baseline understanding of the CAP device’s capability for immediate biofilm disruption. Following this, the double pass treatment achieves a deeper etch with a rate of 2.5–5.5 μm/s, suggesting a more intensive removal process. The triple-pass treatment slightly increases this depth, reaching an etch rate of 2.2–3.7 μm/s, which may indicate the approach’s effectiveness in clearing persistent biofilm remnants. The reduction in the etch rate between the two-pass and three-pass treatments suggests that the biofilms are already completely removed, and the plasma is now acting on the chicken tissue which was found to have a very low etch rate as described above. The five-pass side-by-side treatments (Figure 9), characterized by an etch rate of 2.2–4.3 μm/s and conducted with a 100 μm spacing between passes, results in overlapping regions of effect since a pass would have a 300 μm effect as described above. The five-pass side-by-side treatment results in a larger channel width because the treated channels are arranged next to each other (adjacently). This differs from the previous three treatment configurations, in which a single line is treated repeatedly—for example, in the two-pass treatment, the plasma discharge acts upon a single line twice. It is, however, worth noting that the maximum etching effect occurs within 50–150 μm around the center of the plasma discharge while towards the sides of the plasma discharge, we have less etching effect referred to here as minimum etched depth. This technique broadens the treatment’s impact to an effective width of approximately 800 μm, illustrating how consecutive, strategically spaced treatments can ensure comprehensive coverage of the biofilm-infected area.

**Figure 10.**
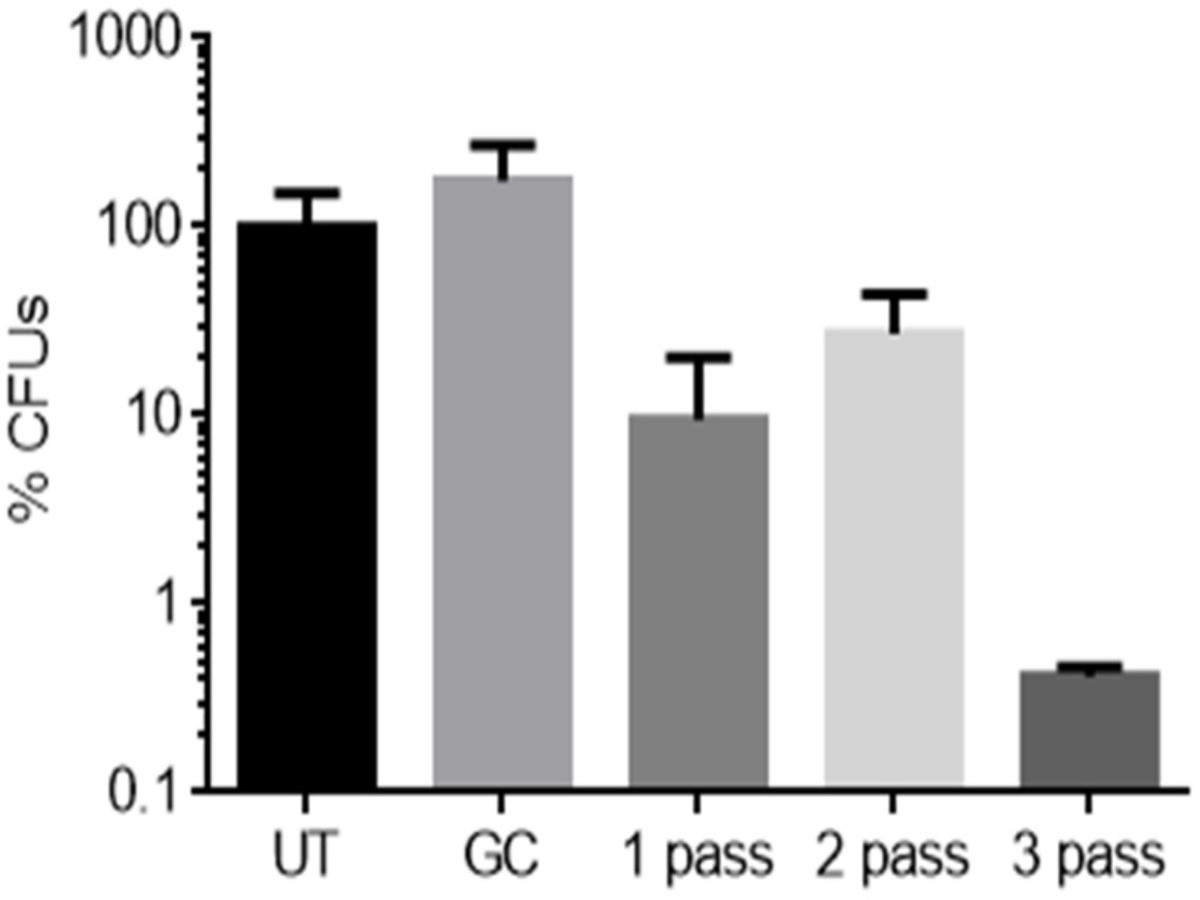
CFU Analysis Results. Triplicate samples were used for the tests. The samples underwent 1-, 2-, and 3pass CAP treatment with the following treatment settings: discharge voltage and current 3.2–3.7 kV, 3 mA, 4 mm sample distance, gas mixture of Ar and H_2_O at 1.5 lpm, and X-Y stage speed of 0.1 mm/s. NB: UT—Untreated, GC—Gas controls.

Figure 10 presents a microbiological analysis of bacterial biofilms on chicken samples, as measured by colony-forming unit (CFU) counts post CAP treatment. The data shows a substantial log reduction in CFU, more than 99% biofilms inactivation following a three-pass CAP treatment carried out on triplicate samples. The one-pass treatment shows about 92% biofilm removal while the two-pass treatment shows an average of 80%. This high percentage of bacterial reduction specifically in the three-pass treatment underlines the efficacy of the CAP device in disinfecting the samples. The one- and two-pass treatments show lower biofilm removal than the three-pass treatments. This directly suggests that during those passes, not all of the biofilm has been removed which is a rationale for more passes for complete biofilm removal. The five-pass side-by-side treatment is not included in this analysis because five-pass treatment is essentially multiple single passes. This result is likely because the biofilm is too thick (~100 μm) for removal in a single pass, as demonstrated in Figure 9. Also, the relative decrease in the two-pass treatment log reduction may suggest issues with the discharge on the samples, probably due to irregularities in the sample topology. One way to improve this would be to read the sample point distance over time as the treatment is being carried out and an algorithm adjusts the sample distance to maintain the desired distance all through the treatment. In our previous work [30], the same treatment technique was used but with a different imaging technique—fluorescence imaging obtained via the autofluorescence of *P. fluorescens.* Using the one-pass treatment, a >99 percent inactivation was already achieved; at two passes, a >99.95% inactivation was achieved; and at three passes, over 99.98% was recorded [29]. This suggests that the treatment guided by autofluorescence performs better than the same treatment guided by TB imaging. This could be due to several reasons such as the TB stain is interfering with the treatment process, the imaging with TB staining is not so accurate as with fluorescence, contaminants are added to the samples via the TB staining, or it could just be an issue with chicken tissue structural variation [29]. However, the most likely scenario is that the biofilm thickness was greater in the TB imaging/removal experiments than in the prior fluorescence guided imaging experiments particularly based on the channel etch experiment. Future efforts are needed to ascertain the reasons due to the differences in the results of these studies [29].

## 4. Conclusions

This study has demonstrated the effectiveness of cold atmospheric pressure (CAP) devices in removing bacterial biofilms from tissue surfaces. Specifically, *P. fluorescens* biofilms grown on chicken tissue, serving as a wound model, were effectively targeted. Chicken tissue was chosen as a substrate for consistency with a related work and ease of access [29] and *P. fluorescens* was selected because of its relatively benign nature and its suitability for comparison with autofluorescence techniques. The use of TB staining, as outlined in our methods, enabled accurate biofilm segmentation, which was analyzed by a computer vision algorithm to optimize the CAP treatment following the methods described here [9]. This image-guided approach represents a precise method for biofilm eradication on tissue surfaces.

Because of the peculiar nature of our study, we could not wait for the biofilm to grow for more than 24 h because, for imaging purposes, we need the biofilm localized to the inoculated region without spreading to the other parts of the chicken surface. Although biofilm thickness measurements could be skewed by irregularity in the chicken surface, efforts were made to flatten out the chicken tissue surface and visual inspection also guided the imaging process. While mature biofilms of *Pseudomonas fluorescens* often require 72 h or more for full development (e.g., on stainless steel surfaces in meat processing environments, where maturity is reached after 5 days with high cell densities > 9.5 log CFU/cm^2^) [34,35], significant early-stage biofilm formation—including attachment, growth, and measurable biomass—can occur within 24 h under optimal conditions, as shown here on polystyrene or stainless steel substrates relevant to food spoilage models [36–38]. This timeframe also aligns with our observations after 24 h, consistent with early but substantial biofilm development on biotic surfaces like chicken tissue, as supported by related studies on Pseudomonas spp. in meat environments where 24 h biofilms are still actively growing but quantifiable [38].

The IV curve of the CAP device, depicted in Figure 4, is particularly informative. It reveals a critical inflection point at approximately 2 kV, where the current sharply increases, indicating plasma activation. The set current and voltage limit is around 3 mA and 4.2 kV, respectively, to allow for the maximum plasma intensity that can be safely reached without damaging the scalpel. Temperature measurements showed the electrode reaching 94.7 °C, indicating direct plasma interaction, while the targeted biofilm area was mildly heated to 36.3 °C. This suggests that biofilm disruption was not due to thermal damage, as the rest of the sample remained at room temperature, highlighting the device’s capability for localized treatment and safety [28].

Furthermore, 3D analysis revealed biofilm thickness on chicken tissues ranging from 20 to 180 μm, consistent with expected thicknesses on mammalian tissues [39–42]. The plasma scalpel achieved an etch rate of 2.2–5.8 μm/s and an impact width of up to 300 μm, allowing for effective overlap in adjacent treatment areas, as seen in Figure 9. These varied treatment types reflect a scalable methodology, from focused, single-pass applications to broader, overlapping treatments. They collectively highlight the CAP device’s flexibility in addressing biofilm of different thicknesses and extents. The overlapping technique show-cases the device’s utility in treating larger biofilm areas effectively, ensuring that no sections of the biofilm are left untreated. Such a nuanced approach is crucial in clinical settings, where the condition of the wound and the extent of biofilm coverage require a customized treatment strategy using the CAP device. CFU analysis after a three-pass treatment showed a reduction in biofilms of approximately 99.94%, underscoring the CAP’s significant biofilm deactivation capability. This level of efficacy compares favorably to other antibacterial methods reported in the literature, such as antimicrobial photodynamic therapy (aPDT), which has demonstrated reductions exceeding 99.9% (often 3–5 log reductions) against bacterial biofilms in in vitro and ex vivo models, including oral and wound-associated biofilms, though aPDT typically requires the application of a photosensitizer like toluidine blue or curcumin for activation [43–45]. The gold standard for removing bacterial biofilms from tissue surfaces in chronic wounds remains mechanical debridement (e.g., sharp or surgical), which physically disrupts and removes biofilms but can be invasive, painful, and incomplete, often achieving only partial reductions without fully eradicating embedded microbes [46]. In contrast, CAP provides a non-contact, non-thermal alternative that has been shown in studies to be non-inferior or even superior to standard best practices in wound healing, with comparable or better bacterial reductions (e.g., >99% in ex vivo and in vivo models) and additional benefits like enhanced granulation and vascularization, while minimizing tissue damage [47–49]. Other methods, such as ultrasound-assisted debridement or enzymatic treatments, typically yield 1–3 log reductions in biofilm bacteria, but CAP’s reactive species offer broader antimicrobial action against multispecies biofilms without promoting resistance [46]. Research groups, including those at the Leibniz Institute for Plasma Science and Technology (Germany) and teams from Ruhr University Bochum, have reported sufficient results with CAP for bacterial and biofilm removal from skin in vitro and ex vivo, achieving up to 5 log reductions in models like burn wounds and dental implants [47–49].

The results of this study suggest the need for future research, possibly increasing the number of passes for total bacterial biofilm eradication and applying this technique in live animal settings. While we acknowledge that additional characterizations, such as confocal laser scanning microscopy (CLSM) with live/dead labeling and scanning electron microscopy (SEM) at various time periods, could further elucidate biofilm inhibition dynamics, the combination of validation techniques from our previous works [9,29]—including fluorescence imaging, CFU reductions, and etch profile analyses—and the 3D microscopic imaging and CFU assessments presented here provide substantial evidence of the CAP’s biofilm removal efficacy. The irregular nature of mammalian skin, and the need to reach areas like corners, suggests the potential use of a robotic arm for more effective treatment. Regarding clinical relevance, this plasma-based approach holds promise for treating chronic wounds by enabling precise, image-guided debridement that reduces bacterial load without damaging underlying tissue, potentially accelerating healing in conditions like diabetic ulcers or burn wounds where biofilms impede recovery. However, practical limitations include the need for specialized equipment (e.g., the plasma scalpel and motorized stage), potential scalability issues for larger wound areas, dependency on accurate TB staining for non-fluorescing biofilms, and the requirement for further in vivo validation to assess long-term safety, tissue response, and efficacy against polymicrobial biofilms in human settings. In conclusion, the study supports the use of CAP devices as a promising method for combatting bacterial biofilms, with significant implications for healthcare and the treatment of infections caused by biofilms if limitations are properly addressed. The precision and efficacy demonstrated here, however, marks a notable advancement in biofilm treatment strategies.

## References

[1] Weigelt MA; McNamara SA; Sanchez D; Hirt PA; Kirsner RS Evidence-Based Review of Antibiofilm Agents for Wound Care. Adv. Wound Care 2021, 10, 13–23. [CrossRef] 10.1089/wound.2020.1193 PMC7698998 32496980

[2] Sen CK Human Wounds and Its Burden: An Updated Compendium of Estimates. Adv. Wound Care 2019, 8, 39–48. [CrossRef] 10.1089/wound.2019.0946 PMC6389759 30809421

[3] Hurlow J; Blanz E; Gaddy JA Clinical investigation of biofilm in non-healing wounds by high resolution microscopy techniques. J. Wound Care 2016, 25, 11–22. [CrossRef] 10.12968/jowc.2016.25.Sup9.S11 PMC5058422 27608736

[4] Malone M; Bjarnsholt T; McBain AJ; James GA; Stoodley P; Leaper D; Tachi M; Schultz G; Swanson T; Wolcott RD The prevalence of biofilms in chronic wounds: A systematic review and meta-analysis of published data. J. Wound Care 2017, 26, 20–25. [CrossRef] 28103163 10.12968/jowc.2017.26.1.20

[5] Farhan N; Jeffery S Utility of MolecuLight i:X for Managing Bacterial Burden in Pediatric Burns. J. Burn Care Res. 2019, 41, 328–338. [CrossRef] 10.1093/jbcr/irz167 31541236

[6] Xu Y; Dhaouadi Y; Stoodley P; Ren D Sensing the unreachable: Challenges and opportunities in biofilm detection. Curr. Opin. Biotechnol. 2020, 64, 79–84. [CrossRef] 31766008 10.1016/j.copbio.2019.10.009 PMC7242124

[7] Rennie M; Dunham D; Lindvere-Teene L; Raizman R; Hill R; Linden R Understanding Real-Time Fluorescence Signals from Bacteria and Wound Tissues Observed with the MolecuLight i:XTM. Diagnostics 2019, 9, 22. [CrossRef] 30813551 10.3390/diagnostics9010022 PMC6468690

[8] Pijpe A; Ozdemir Y; Sinnige JC; Kwa KAA; Middelkoop E; De Vries AM Detection of bacteria in burn wounds with a novel handheld autofluorescence wound imaging device: A pilot study. J. Wound Care 2019, 28, 548–554. [CrossRef] 31393796 10.12968/jowc.2019.28.8.548

[9] Okebiorun M; Oberbeck C; Waite C; Clark S; Miller D; Smith EHB; Cornell KA; Browning J Selective Optical Imaging for Detection of Bacterial Biofilms in Tissues. J. Imaging 2023, 9, 160. [CrossRef] 37623692 10.3390/jimaging9080160 PMC10455256

[10] Dada VK; Sharma N; Sudan R; Sethi H; Dada T; Pangtey MS Anterior capsule staining for capsulorhexis in cases of white cataract: Comparative clinical study. J. Cataract. Refract. Surg. 2004, 30, 326–333. [CrossRef] 15030820 10.1016/S0886-3350(03)00573-X

[11] Kuhn F Chromovitrectomy. In Vitreoretinal Surgery: Strategies and Tactics; Springer International Publishing AG: Cham, Switzerland, 2016; pp. 325–330. [CrossRef]

[12] Jacob S; Agarwal A; Agarwal A; Agarwal S; Chowdhary S; Chowdhary R; Bagmar AA Trypan blue as an adjunct for safe phacoemulsification in eyes with white cataract. J. Cataract. Refract. Surg. 2002, 28, 1819–1825. [CrossRef] 12388035 10.1016/s0886-3350(01)01316-5

[13] Nanavaty MA; Johar K; Sivasankaran MA; Vasavada AR; Praveen MR; Zetterström C Effect of trypan blue staining on the density and viability of lens epithelial cells in white cataract. J. Cataract. Refract. Surg. 2006, 32, 1483–1488. [CrossRef] 16931259 10.1016/j.jcrs.2006.04.017

[14] Farah ME; Maia M; Furlani B; Bottós J; Meyer CH; Lima V; Penha FM; Costa EF; Rodrigues EB Current Concepts of Trypan Blue in Chromovitrectomy; Karger Medical and Scientific Publishers: Basel, Switzerland, 2008; Volume 42, pp. 91–100. [CrossRef] 10.1159/000138980 18535383

[15] Chang YS; Tseng SY; Tseng SH Comparison of dyes for cataract surgery. Part 2: Efficacy of capsule staining in a rabbit model. J. Cataract. Refract. Surg. 2005, 31, 799–804. [CrossRef] 15899459 10.1016/j.jcrs.2004.09.029

[16] Lou B-S; Lai C-H; Chu T-P; Hsieh J-H; Chen C-M; Su Y-M; Hou C-W; Chou P-Y; Lee J-W Parameters Affecting the Antimicrobial Properties of Cold Atmospheric Plasma Jet. J. Clin. Med. 2019, 8, 1930. [CrossRef] 31717600 10.3390/jcm8111930 PMC6912271

[17] Jang JY; Hong YJ; Lim J; Choi JS; Choi EH; Kang S; Rhim H Cold atmospheric plasma (CAP), a novel physicochemical source, induces neural differentiation through cross-talk between the specific RONS cascade and Trk/Ras/ERK signaling pathway. Biomaterials 2018, 156, 258–273. [CrossRef] 29222974 10.1016/j.biomaterials.2017.11.045

[18] Cornell KA; White A; Croteau A; Carlson J; Kennedy Z; Miller D; Provost M; Goering S; Plumlee D; Browning J Fabrication and Performance of a Multidischarge Cold-Atmospheric Pressure Plasma Array. IEEE Trans. Plasma Sci. 2021, 49, 1388–1395. [CrossRef] 10.1109/TPS.2021.3064993 PMC8132946 34024956

[19] Puliyalil H; Cvelbar U Selective Plasma Etching of Polymeric Substrates for Advanced Applications. Nanomaterials 2016, 6, 108. [CrossRef] 28335238 10.3390/nano6060108 PMC5302619

[20] Pereira S; Pinto E; Ribeiro PA; Sério S Study of a Cold Atmospheric Pressure Plasma jet device for indirect treatment of Squamous Cell Carcinoma. Clin. Plasma Med. 2019, 13, 9–14. [CrossRef]

[21] Bosch LT; Habedank B; Siebert D; Mrotzek J; Viöl W Cold Atmospheric Pressure Plasma Comb—A Physical Approach for Pediculosis Treatment. Int. J. Environ. Res. Public Health 2019, 16, 19. [CrossRef] 10.3390/ijerph16010019 PMC6338894 30577656

[22] Jampa-ngern S; Viravaidya-Pasuwat K; Suvanasuthi S; Khantachawana A Effect of laser diode light irradiation on growth capability of human hair follicle dermal papilla cells. In Proceedings of the 2017 39th Annual International Conference of the IEEE Engineering in Medicine and Biology Society (EMBC), IEEE, Jeju Island, Republic of Korea, 11–15 July 2017; pp. 3592–3595. [CrossRef] 10.1109/EMBC.2017.8037634 29060675

[23] Balzer J; Demir E; Kogelheide F; Fuchs PC; Stapelmann K; Opländer C Cold atmospheric plasma (CAP) differently affects migration and differentiation of keratinocytes via hydrogen peroxide and nitric oxide-related products. Clin. Plasma Med. 2019, 13, 1–8. [CrossRef]

[24] Semmler ML; Bekeschus S; Schäfer M; Bernhardt T; Fischer T; Witzke K; Boeckmann L Molecular mechanisms of the efficacy of cold atmospheric pressure plasma (CAP) in cancer treatment. Cancers 2020, 12, 269. [CrossRef] 31979114 10.3390/cancers12020269 PMC7072164

[25] Saadati F; Mahdikia H; Abbaszadeh HA; Abdollahifar MA; Khoramgah MS; Shokri B Comparison of Direct and Indirect cold atmospheric-pressure plasma methods in the B_16_F_10_ melanoma cancer cells treatment. Sci. Rep. 2018, 8, 7689. [CrossRef] 29769707 10.1038/s41598-018-25990-9 PMC5955918

[26] Racka-Szmidt K; Stonio B; Żelazko J; Filipiak M; Sochacki M A Review: Inductively Coupled Plasma Reactive Ion Etching of Silicon Carbide. Materials 2021, 15, 123. [CrossRef] 35009277 10.3390/ma15010123 PMC8745874

[27] Kos S; Blagus T; Cemazar M; Filipic G; Sersa G; Cvelbar U; Yousfi M Safety aspects of atmospheric pressure helium plasma jet operation on skin: In vivo study on mouse skin. PLoS ONE 2017, 12, e0174966. [CrossRef] 10.1371/journal.pone.0174966 PMC5381889 28379998

[28] Kalghatgi S; Kelly CM; Cerchar E; Torabi B; Alekseev O; Fridman A; Friedman G; Azizkhan-Clifford J; Koutsopoulos S Effects of Non-Thermal Plasma on Mammalian Cells. PLoS ONE 2011, 6, e16270. [CrossRef] 10.1371/journal.pone.0016270 PMC3025030 21283714

[29] Okebiorun MO; Oberbeck C; Waite C; Clark S; Alomar Z; Miller D; Cornell K; Browning J Autofluorescence-Guided Removal of Bacterial Biofilms from Tissues Using Cold Atmospheric Pressure Plasma (CAP). IEEE Trans. Radiat. Plasma Med. Sci. 2024, 8, 990–996. [CrossRef] 39512890 10.1109/trpms.2024.3370503 PMC11540416

[30] Browning J; Cornell K Plasma Scalpel for Selective Removal of Microbes and Microbial Biofilms. US Patent 1187198, 2024. Available online: https://www.patentdigest.org/patent/?patent_id=11871978 (accessed on 8 July 2025).

[31] Okebiorun M; Waite C; May H; Miller D; Cornell K; Browning J Wound-Based Bacterial Biofilm Imaging for Selective Cold Atmospheric Pressure Plasma Treatment. In Proceedings of the 2021 IEEE International Conference on Plasma Science (ICOPS), Lake Tahoe, NV, USA, 12–16 September 2021. [CrossRef]

[32] Malecha K; Golonka LJ Microchannel fabrication process in LTCC ceramics. Microelectron. Reliab. 2008, 48, 866–871. [CrossRef]

[33] Cornell KA; Benfield K; Berntsen T; Clingerman J; Croteau A; Goering S; Moyer D; Provost M; White A; Plumlee D; A Cold Atmospheric Pressure Plasma Discharge Device Exerts Antimicrobial Effects. Int. J. Latest Trends Eng. Technol. 2020, 15, 36–41. [PubMed Central] PMC7098701 32219149

[34] Borremans A; Lenaerts S; Crauwels S; Lievens B; Van Campenhout L Biofilm Formation by Meat-Borne Pseudomonas fluorescens on Stainless Steel and Its Resistance to Sanitizers. Food Control 2018, 91, 397–403. [CrossRef]

[35] Cruz KL; de Souza A Characterization of Biofilm Production by *Pseudomonas fluorescens* Isolated from Refrigerated Raw Buffalo Milk. J. Food Sci. Technol. 2019, 56, 4595–4604. [CrossRef] 31686691 10.1007/s13197-019-03924-1 PMC6801270

[36] Chen X; Thomsen TR; Winkler H; Xu Y Influence of Biofilm Growth Age, Media, Antibiotic Concentration and Exposure Time on Staphylococcus aureus and Pseudomonas aeruginosa Biofilm Removal In Vitro. BMC Microbiol. 2020, 20, 264. [CrossRef] 32831025 10.1186/s12866-020-01947-9 PMC7444035

[37] Angarano V; Smet C; Akkermans S; Akritidou T; Huyck B; Chieffi A; Van Impe JFM A Reproducible Method for Growing Biofilms on Polystyrene Surfaces: Biomass and Bacterial Viability Evolution of *Pseudomonas fluorescens* and *Staphylococcus epidermidis*. Appl. Sci. 2020, 10, 4544. [CrossRef]

[38] Calhoun C; Geornaras I; Zhang P Pseudomonas in Meat Processing Environments. Foods 2025, 14, 1615. [CrossRef] 40361697 10.3390/foods14091615 PMC12071725

[39] Hou J; Wang C; Rozenbaum RT; Gusnaniar N; de Jong ED; Woudstra W; Geertsema-Doornbusch GI; Atema-Smit J; Sjollema J; Ren Y; Bacterial Density and Biofilm Structure Determined by Optical Coherence Tomography. Sci. Rep. 2019, 9, 9794. [CrossRef] 31278369 10.1038/s41598-019-46196-7 PMC6611762

[40] Murga R; Stewart PS; Daly D Quantitative analysis of biofilm thickness variability. Biotechnol. Bioeng. 1995, 45, 503–510. [CrossRef] 18623250 10.1002/bit.260450607

[41] Zhao G; Usui ML; Lippman SI; James GA; Stewart PS; Fleckman P; Olerud JE Biofilms and Inflammation in Chronic Wounds. Adv. Wound Care 2013, 2, 389–399. [CrossRef] 10.1089/wound.2012.0381 PMC3763221 24527355

[42] Wilson C; Lukowicz R; Merchant S; Valquier-Flynn H; Caballero J; Sandoval J; Okuom M; Huber C; Brooks TD; Wilson E; Quantitative and Qualitative Assessment Methods for Biofilm Growth: A Mini-Review. Res. Rev. J. Eng. Technol. 2017, 6. Available online: http://www.rroij.com/open-access/quantitative-and-qualitative-assessment-methods-for-biofilm-growth-a-minireview-.pdf (accessed on 8 July 2025). PMC6133255 30214915

[43] Sun Y; Ogawa R; Xiao B; Feng Y; Wu Y; Chen L; Gao X; Chen H Antimicrobial photodynamic therapy in skin wound healing: A systematic review of animal studies. Int. Wound J. 2020, 17, 285–299. [CrossRef] 31724831 10.1111/iwj.13269 PMC7948698

[44] de Melo WCMA; Celiešiūtė-Germanienė R; Šimonis P; Stirkė A Antimicrobial Photodynamic Therapy (aPDT) for Biofilm Treatments. Possible Synergy Between aPDT and Pulsed Electric Fields. Virulence 2021, 12, 2247–2272. [CrossRef] 34496717 10.1080/21505594.2021.1960105 PMC8437467

[45] Hu X; Huang Y-Y; Wang Y; Wang X; Hamblin MR Antimicrobial Photodynamic Therapy to Control Clinically Relevant Biofilm Infections. Front. Microbiol. 2018, 9, 1299. [CrossRef] 29997579 10.3389/fmicb.2018.01299 PMC6030385

[46] Liu Y; Long S; Wang H; Wang Y Biofilm Therapy for Chronic Wounds. Int. Wound J. 2024, 21, e14667. [CrossRef] 10.1111/iwj.14667 PMC10858329 38339793

[47] Matthes R; Jablonowski L; Pitchika V; Holtfreter B; Eberhard C; Seifert L; Gerling T; Scholten LV; Schlüter R; Kocher T Efficiency of Biofilm Removal by Combination of Water Jet and Cold Plasma: An In-Vitro Study. BMC Oral Health 2022, 22, 157. [CrossRef] 35524324 10.1186/s12903-022-02195-1 PMC9074283

[48] Matthes R; Jablonowski L; Miebach L; Pitchika V; Holtfreter B; Eberhard C; Seifert L; Gerling T; Schlüter R; Kocher T; In-Vitro Biofilm Removal Efficacy Using Water Jet in Combination with Cold Plasma Technology on Dental Titanium Implants. Int. J. Mol. Sci. 2023, 24, 1606. [CrossRef] [PubMed] 36675120 10.3390/ijms24021606 PMC9867126

[49] Abu Rached N; Kley S; Storck M; Meyer T; Stücker M Cold Plasma Therapy in Chronic Wounds—A Multicenter, Randomized Controlled Clinical Trial (Plasma on Chronic Wounds for Epidermal Regeneration Study): Preliminary Results. J. Clin. Med. 2023, 12, 5121. [CrossRef] 37568525 10.3390/jcm12155121 PMC10419810

